# Predictive value of pediatric respiratory-induced diaphragm motion quantified using pre-treatment 4DCT and CBCTs

**DOI:** 10.1186/s13014-018-1143-6

**Published:** 2018-10-11

**Authors:** Sophie C. Huijskens, Irma W. E. M. van Dijk, Jorrit Visser, Brian V. Balgobind, Coen R. N. Rasch, Tanja Alderliesten, Arjan Bel

**Affiliations:** Department of Radiation Oncology, Cancer Center Amsterdam, Amsterdam UMC, University of Amsterdam, Office Z1-217, Meibergdreef 9, 1105 AZ Amsterdam, The Netherlands

**Keywords:** Respiratory-induced motion, Pediatric RT, IGRT, 4DCT

## Abstract

**Background:**

In adults, a single pre-treatment four-dimensional CT (4D-CT) acquisition is often used to account for respiratory-induced target motion during radiotherapy. However, studies have indicated that a 4D-CT is not always representative for respiratory motion. Our aim was to investigate whether respiratory-induced diaphragm motion in children on a single pre-treatment 4DCT can accurately predict respiratory-induced diaphragm motion as observed on cone beam CTs (CBCTs).

**Methods:**

Twelve patients (mean age 14.5 yrs.; range 8.6–17.9 yrs) were retrospectively included based on visibility of the diaphragm on abdominal or thoracic imaging data acquired during free breathing. A 4DCT for planning purposes and daily/weekly CBCTs (total 125; range 4–29 per patient) acquired prior to dose delivery were available. The amplitude, corresponding to the difference in position of the diaphragm in cranial-caudal direction in end-inspiration and end-expiration phases, was extracted from the 4DCT (A_4DCT_). The amplitude in CBCTs (A_CBCT_) was defined as displacement between averaged in- and expiration diaphragm positions on corresponding projection images, and the distribution of A_CBCT_ was compared to A_4DCT_ (one-sample t-test, significance level *p <* 0.05).

**Results:**

Over all patients, the mean A_4DCT_ was 10.4 mm and the mean A_CBCT_ 11.6 mm. For 9/12 patients, A_4DCT_ differed significantly (*p* < 0.05) from A_CBCT_. Differences > 3 mm were found in 69/125 CBCTs (55%), with A_4DCT_ mostly underestimating A_CBCT_. For 7/12 patients, diaphragm positions differed significantly from the baseline position.

**Conclusion:**

Respiratory-induced diaphragm motion determined on 4DCT does not accurately predict the daily respiratory-induced diaphragm motion observed on CBCTs, as the amplitude and baseline position differed statistically significantly in the majority of patients. Regular monitoring of respiratory motion during the treatment course using CBCTs could yield a higher accuracy when a daily adaptation to the actual breathing amplitude takes place.

**Electronic supplementary material:**

The online version of this article (10.1186/s13014-018-1143-6) contains supplementary material, which is available to authorized users.

## Background

Respiratory motion during radiotherapy may lead to uncertainties in radiation dose delivery, and accounting for it is challenging. Continuous efforts in the field have led to innovative methods to deal with respiratory motion, such as breath holding, beam gating or tracking, or online visualizing of respiratory motion (e.g., magnetic resonance (MR) guidance) [1]. These techniques increase treatment time and clinical workload, and often require patient training which might lead to additional patient distress and anxiety. Although children could also benefit from these techniques [2, 3], it is known that they experience radiotherapy already as a stressful procedure [4–6], and therefore the use of these techniques remains limited in pediatric radiotherapy.

Respiratory motion is more commonly accounted for by the use of an internal margin (IM) that encompasses the clinical target volume (CTV), defining an internal target volume (ITV) [7]. This leads to unfavourable large margins, thereby increasing dose to surrounding healthy tissues. The mid-ventilation based planning target volume (PTV) approach accounts for both respiratory motion and day-to-day geometrical variations and achieves smaller margins [8–10]. No matter which approach is used, in order to assess respiratory motion, a pre-treatment respiratory-correlated four-dimensional computed tomography (4DCT) is essential.

In adults, a single pre-treatment 4DCT is used extensively to assess respiratory motion [11–13]. Our previous study showed that respiratory-induced diaphragm motion throughout the treatment course was more stable in children than previously reported by others in adults [14]. This implies that a single measurement could be more representative in children than in adults and suggests that a pre-treatment 4DCT in children could be at least equally beneficial as it is in adults [14]. To our knowledge, only few institutes have clinically introduced 4DCT for pediatric radiotherapy planning purposes and reported on this [15–17]. Generally, the conclusion was that 4DCT is an effective tool to accurately determine respiratory-induced organ motion (e.g., liver, spleen, kidneys) for pediatric specific cases, providing the data on respiratory motion needed to define margins, thereby stressing the need for individualized margins [15–17]. However, studies on adult patients have also indicated that respiratory motion, as measured on 4DCT, is not always representative for respiratory motion during the subsequent treatment course [11–13, 18]. Therefore, a single pre-treatment measurement for planning purposes might be a misrepresentation and could lead to under- or overestimating respiratory motion, yielding insufficient target coverage or undesired dose to organs at risk (OARs) [19]. In previous pediatric studies, respiratory-induced organ motion was only measured within a single 4DCT per patient [15–17], without assessing how representative the 4DCT is for respiratory-induced motion during the treatment course. To assess the daily respiratory-induced motion during the treatment course in children, cone beam CT (CBCT) scans acquired for position verification can be used [14]. With increasing use of 4DCT in pediatric radiotherapy, assessment of the predictive value of the measurements on 4DCT is essential to take full advantage of this technique.

Therefore, the aim of this study was to investigate if respiratory-induced diaphragm motion during radiotherapy in children, as a surrogate for respiratory-induced abdominal motion, can be accurately predicted by a single measurement based on a pre-treatment 4DCT. We also analyzed possible time trends in respiratory-induced diaphragm motion over the complete treatment course. Finally, to investigate if measurements on CBCTs could be predictive for respiratory-induced diaphragm motion that continues post-acquisition (i.e., the actual respiratory motion during dose delivery), we quantified and compared respiratory-induced diaphragm motion on two CBCTs acquired within one treatment session with an interval of minutes.

## Methods

### Patient data

From November 2014 to December 2017, fourteen patients (mean age 14.5 years, range 8.6–17.9 years) had a 4DCT scan during free breathing for treatment planning purposes and multiple CBCT scans acquired for position verification during the treatment course. Patients were included when the complete diaphragm was visible on the 4DCT and CBCTs. Two of the fourteen eligible patients were excluded from this retrospective study; one patient received general anaesthesia (GA) and the imaging data of another patient showed severe motion artefacts. Patient characteristics are listed in Table 1.

**Table 1:** Patient characteristics

No.	Sex	Tumor type	Age at 4DCT (years)	Height (cm)	Weight (kg)	No. of CBCTs
1	M	Ewingsarcoma	10.7	137	28.0	7
2	F	Ewingsarcoma	16.3	162	66.5	8
3^a^	F	Ewingsarcoma	17.9	163	52.6	12
4^a^	M	Osteosarcoma	14.9	186	71.6	5
5^b^	F	Ewingsarcoma	12.5	151	70.0	29
6^b^	M	ERMS	16.1	182	57.6	19
7^b^	M	Ewingsarcoma	14.3	182	55.6	6
8	M	CCS	8.6	125	23.0	5
9	F	Non Hodgkin	17.1	178	86.0	11
10	F	Ewingsarcoma	14.8	153	57.0	7
11^b^	M	RMS prostate	16.7	186	64.0	4
12	M	Non-RMS	14.4	172	59.0	12

Abbreviations: *M* male, *F* female, *4DCT* four-dimensional computed tomography, *CBCT* cone beam CT, *(E)RMS* embryonal rhabdomyosarcoma, *CCS* clear cell sarcoma

^a^Patients had within multiple treatment sessions repetitive CBCTs (only the 1st and 2nd were included in the analysis)

^b^Patients had two CBCTs within one treatment session

### 4DCT

**Fig. 1:**
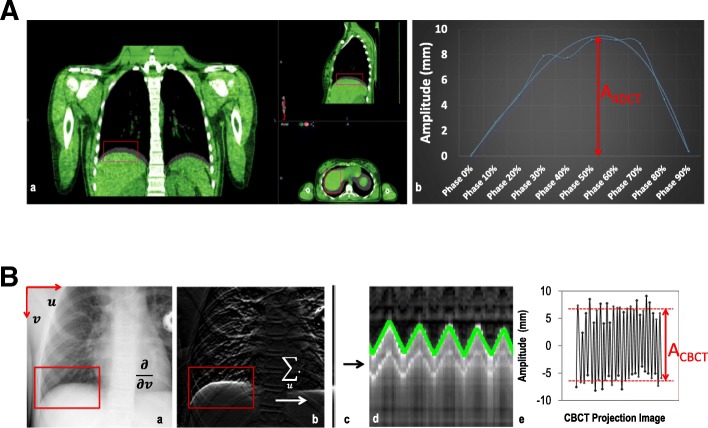
**a** Left) Rigid registration of the cranial-caudal position of the right diaphragm (inside the red box) in all breathing phases. Right) The difference between the most extreme translations was defined as the amplitude (A_4DCT_). **b** Amsterdam Shroud (AS) method to manually track the CC diaphragm position in CBCT projection images. a) Region of interest (red box). b) CC gradient filter applied and sum of all pixels creates a 1D image. C) This is repeated for all projection images, creating a 2D image. d) Detection of diaphragm positions in inhale and exhale breathing phases. e) Pixel coordinates translated to CC position. The amplitude was defined as the displacement between averaged end-inspiration and end-expiration diaphragm positions (A_CBCT_)

The 4DCTs (LightSpeed RT16 system, General Electric Company, Waukesha WI, USA) were acquired during free breathing (using the Varian RPM system v1.7.3). The respiratory cycle was divided into ten phase bins, resulting in ten phase scans (slice thickness 2.5 mm). Velocity (Velocity, version 3.1, Varian Medical Systems, Palo Alto, CA, USA) was used to perform a two-step rigid registration. The end-inspiration phase scan (i.e., the 0% phase scan) was used as a reference and was registered to the other nine phase scans. In one patient (no. 7), the 90% phase scan served as the reference, due to motion artefacts on the 0% phase scan. For all other patients and phase scans, no (severe) motion artefacts were seen that could have hampered the registrations. The right diaphragm domes were matched manually in the cranial-caudal (CC) direction, using translations only. The obtained translations resulted in the excursion of the right diaphragm dome throughout the respiratory cycle in the CC direction. The difference between the most extreme translations, typically the 0% to the 50% or 60% phase scan, was defined as the amplitude (A_4DCT_) (Fig. 1a)_._

### CBCT

For each patient, CBCT scans during free breathing (Synergy, Elekta Oncology systems, Crawly, UK) for position verification were daily and/or weekly acquired according to a customized extended no-action level (eNAL) protocol [20], totalling 125 CBCT scans (range 4–29 per patient). Six of the 12 patients had multiple CBCTs within one treatment session (total 13, range 2–5, not included in the 125), depending on their treatment protocol (e.g., stereotactic or spinal cord irradiation), or in one case a second CBCT was necessary due to artefacts. These artefacts, however, still allowed sufficient number of useable projection images for evaluation of respiratory-induced diaphragm motion. For all CBCTs, a single projection was acquired in 180 ms and the energy was 120 kV, tube current 10 mA and 10 or 40 ms exposure time per projection. The circumferential rotation varied from 200 to 360 degrees and the acquisition time varied between 35 s and 120 s, resulting in a variation in number of projection images per CBCT (180 to 760).

The methodology to extract the respiratory-induced diaphragm motion has been described previously [14]. In short, for each CBCT, an adapted version of the Amsterdam Shroud (AS) method was used to create an AS image [21], allowing for manual selection of the projection images corresponding to the end-inspiration and end-expiration positions of the right diaphragm dome. In each of those selected projection images, we then manually determined the CC position of the top of the right diaphragm dome. Pixel coordinates were corrected for the scanner geometry and translated relative to the patients’ isocenter [22]. This resulted in a patient- and CBCT-dependent timeframe describing the CC position of the diaphragm in end-inspiration and end-expiration phases (peaks) over the course of CBCT acquisition. The amplitude was defined as the displacement between averaged end-inspiration and averaged end-expiration diaphragm positions (A_CBCT_) (Fig. 1b).

### Statistical analysis

In order to determine whether respiratory-induced diaphragm motion observed in a single pre-treatment 4DCT accurately predicted respiratory-induced diaphragm motion during the treatment course, we tested for each patient separately whether A_CBCT_ over all CBCTs differed from A_4DCT_ using a one-sample t-test (significance level *p < 0.05*). For each patient, we calculated the absolute differences between A_4DCT_ and each A_CBCT_, and determined for which fractions the difference was larger than 3 mm (4DCT slice thickness is 2.5 mm).

To investigate possible time trends for each patient, we applied a linear regression analysis on A_CBCT_ over the course of treatment. We also calculated the interfractional variability of A_CBCT_ per patient (i.e., the SD over A_CBCT_). The diaphragm position on the averaged pre-treatment 4DCT (this is the averaged scan, based on all phases of the 4DCT) was considered as a reference (i.e., baseline), for which a rigid registration on bony anatomy was taken into account. We then calculated for each patient separately shifts of the diaphragm position in CC direction (i.e., the average position of the diaphragm during one CBCT) over the course of treatment. We used a one-sample t-test to test whether diaphragm positions on CBCT significantly differed from the baseline diaphragm position on the averaged 4DCT.

Additionally, six patients received multiple CBCTs during one fraction. For above mentioned analysis, only the first CBCT was included in the analysis. To validate if respiratory-induced diaphragm motion measurements on CBCTs could be predictive for respiratory-induced motion that continues post-acquisition (i.e., the actual respiratory motion during dose delivery), we compared the amplitude measured on the first and second CBCT (A_CBCT(1)_ to A_CBCT(2)_; paired t-test, significance level *p < 0.05*). These are acquired within 4–10 min, which is a representative time interval between CBCT acquisition and start of dose delivery.

R Software package version 3.2.1. (R foundation for statistical Computing, Austria) was used for all statistical analysis.

## Results

**Fig. 2:**
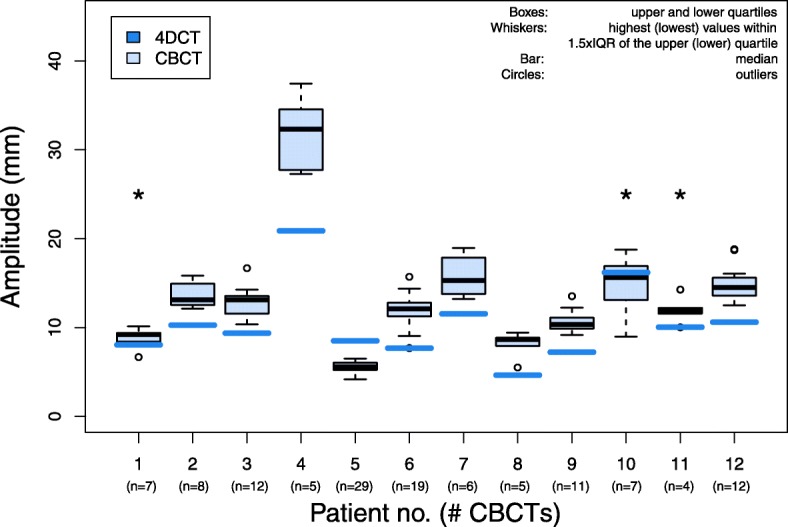
Respiratory-induced diaphragm motion on 4DCT (blue solid lines) and CBCT data (boxplots) of 12 children during image-guided radiotherapy. Patients with * showed no significant differences (*p > 0.05*)

Over all patients, the mean A_4DCT_ was 10.4 mm (SD = 4.3 mm) and the mean A_CBCT_ was 11.6 mm (SD = 5.7 mm). For 9 out of 12 patients, A_4DCT_ differed statistically significantly (*p <* 0.05) from A_CBCT_ (Fig. 2). Underestimation of A_4DCT_ compared to A_CBCT_ was found in 76% of the measurements (95/125 CBCTs), and was observed in 11 out of 12 patients. Hence, overestimation was found in 24% of the measurements (30/125 CBCTs), and was observed in 3 out of 12 patients. Differences > 3 mm were found in 69 of the 125 CBCTs (55%).

For each patient, we plotted A_CBCT_ over time of the treatment course (Additional file 1: Figure S1) where day 0 is the day of 4DCT acquisition. We found that 8 of the 12 trend lines had a negative slope. Absolute slopes larger than 0.1 mm/day were observed in 4 patients with only few data points (4 to 6 points for patients 4, 7, 8, and 11). For the other patients, with more data points, we observed by both visual inspection and linear fits, no obvious time trend (absolute slopes ranged from 0.00–0.09 mm/day).

**Fig. 3:**
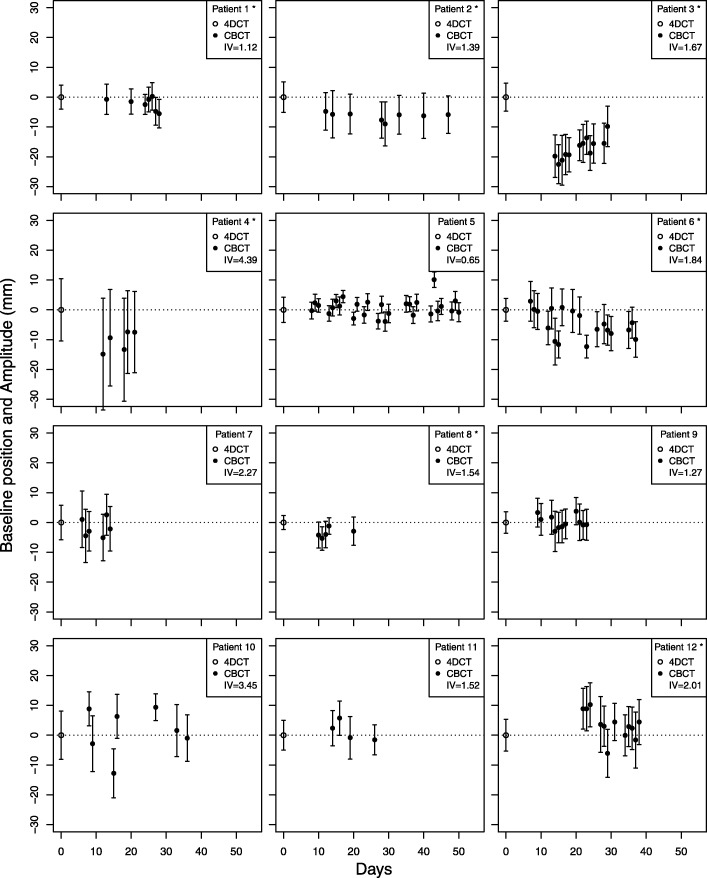
Open dots and dashed lines represent the baseline diaphragm position on the planning 4DCT (averaged 4DCT). Black dots represent the average positions of the diaphragm on each CBCT. Whiskers represent measured end-inspiration and end-expiration positions on 4DCT and CBCTs for each patient plotted as function of days. Day 0 is the day of 4DCT acquisition (open dots), IV = interfractional variability, * indicates a significant difference from baseline (*p* < 0.05)

Overall, interfractional variability of A_CBCT_ was 2.2 mm (range 0.7–4.4 mm; individual values shown in Fig. 3). For 7 out of 12 patients, averaged diaphragm positions in CC direction observed on CBCTs differed statistically significantly (mean 7.4 mm, SD = 5.9 mm; *p <* 0.05) from the baseline diaphragm position as measured on the averaged 4DCT (Fig. 3).

**Fig. 4:**
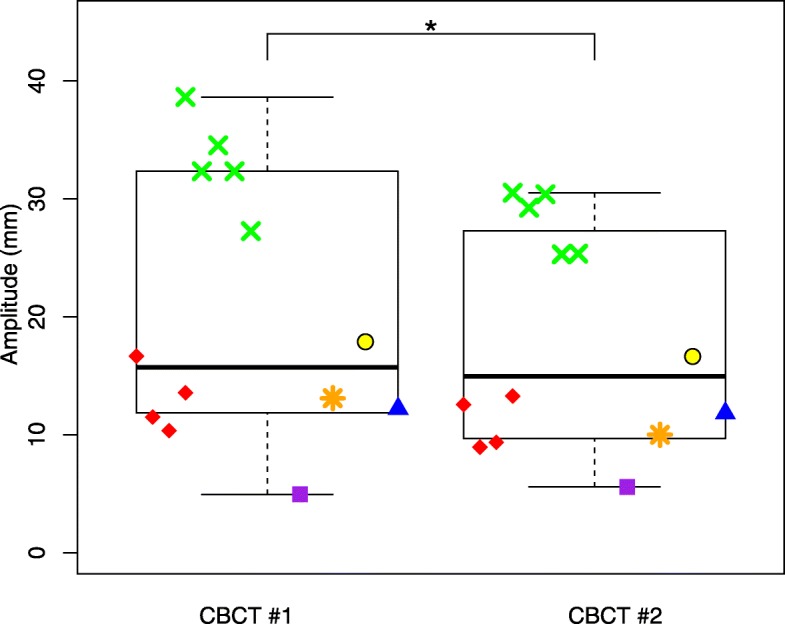
Significant difference (indicated by *) between amplitudes measured on the first and second CBCT acquired within one treatment session. Each different color and symbol represent different patients

Patients 3 and 4 had on multiple days additional CBCT scans. Patients 5, 6, 7, and 11 had only on one day an additional CBCT. The subsequent CBCTs were acquired within a time interval of 4–10 min. Over all six patients, A_CBCT(2)_ was significantly different from A_CBCT(1)_ (mean difference 2.9 mm, SD = 2.5 mm, *p* = 0.002) (Fig. 4). However, patient 4 (in Fig. 4 indicated by the green cross symbol) showed significant deviations from the group measurements. We performed a sensitivity analysis by excluding this patient from the analysis. Although the average difference was now 1.7 mm (SD = 1.4 mm), A_BCT(2)_ remained significantly different from A_CBCT(1)_ (*p* = 0.033).

## Discussion

In this study we investigated if respiratory-induced diaphragm motion in children during radiotherapy could be accurately predicted based on a 4DCT scan acquired prior to the start of radiotherapy. We compared the amplitude of the diaphragm displacement on 4DCT and daily/weekly CBCTs, enabling an encompassing analysis of pre-treatment respiratory-induced motion and during complete radiotherapy courses in children. This also enabled to investigate possible time trends and day-to-day variations. Our study showed that for the majority of patients (9/12 patients) respiratory-induced diaphragm motion on 4DCT differed significantly from measurements on CBCTs. Also, respiratory-induced diaphragm motion derived from CBCTs acquired within an interval of minutes was statistically significantly different. No obvious time trends in respiratory-induced diaphragm motion over the course of treatment were found, but significant baseline shifts of the diaphragm position were seen in 7/12 patients. These findings suggest that respiratory-induced diaphragm motion as measured on 4DCT was not representative for respiratory motion during the treatment course.

Although acquisition of 4DCT and CBCT scans differs, the amplitude was quantified in a similar way. During 4DCT acquisition, one breathing cycle is included per table position. This represents only a short time period, and amplitude between consecutive breathing cycles varies [14]. This uncertainty would be of a similar size (2.2 mm) as measured during the consecutive CBCTs. Additionally, a 4DCT scan is binned into 10 3D-breathing-phase scans corresponding to 10 phases of the respiratory cycle using phase binning, which already underestimates the diaphragm motion slightly [23]. For 4DCT, we quantified the amplitude as the maximal displacement between the most extreme diaphragm positions. On the other hand, CBCT acquisition time varied between 35 and 120 s and thus included more breathing cycles compared to the 4DCT. In the amplitude calculations for both types of CT scans, we averaged the diaphragm positions on the CBCT scan for in- and exhale phases and the difference between these averaged inhale and exhale diaphragm position defined the amplitude on CBCT. This is a slightly different approach as used by others [13, 24], who binned projection images corresponding to in- and exhale phases for (4D-) CBCT reconstructions, thereby averaging (e.g. blurring) the actual diaphragm positions on the reconstructed image. While different approaches have their advantages and limitations, for the comparison of 4DCT and CBCT data, in this study we chose to average the actual diaphragm positions at end-inspiration and end-expiration as measured on the corresponding projection image. This guarantees that all projection images are taken into account, and represents a realistic view of the actual motion happened.

Since we used respiratory-induced diaphragm motion as a surrogate for respiratory-induced abdominal motion, our outcomes cannot be directly applied for calculating safety margins. This was shown by Panandiker et al. who assessed intrafractional renal and diaphragm motion on free-breathing 4DCTs in 20 children, and concluded that measuring diaphragm motion alone does not reliably quantify renal motion [15]. Adult studies reported both positive and negative on using the diaphragm as a reliable surrogate for tumor or organ motion [25–27]. Two other pediatric studies have reported on intrafractional abdominal organ and tumor motion using 4DCT scans and concluded that 4DCT is an effective tool to accurately determine respiratory-induced organ motion for pediatric specific cases, leading to the desired more individualized treatment approach [16, 17]. However, in these studies, correlations of respiratory-induced organ motion with diaphragm motion were not investigated. Since respiratory-induced diaphragm motion does not necessarily correlate with tumor motion, using the diaphragm as a surrogate for abdominal and thoracic organ motion could induce some inaccuracies and uncertainties that need to be taken into account for treatment planning purposes.

A 4DCT involves a slightly higher imaging dose compared to a 3DCT and due to the ALARA principle (keeping doses as low as reasonably achievable) and previously reported radiation risks in children from CT scans [28–30], reluctance remains to use 4DCT in the pediatric population. It would be interesting to investigate the possible correlation between external thorax vertical displacement and the internal longitudinal diaphragm motion in children. In case of a strong and clear correlation, which was found for adults [31], the possibility of using an external reliable surrogate for internal respiratory-induced organ motion could decrease additional imaging dose. Since daily imaging dose adds to the total treatment dose, minimizing additional dose has to be carefully considered. Ultimately, the additional imaging dose in the pediatric population should be balanced with better treatment planning and delivery, in order to minimize dose to the healthy surrounding tissues. Especially, 4DMRI shows to be a promising tool for future image- and MR-guided pediatric radiotherapy, providing superior soft tissue contrast and higher resolution in CC direction, while avoiding ionizing radiation doses [32, 33].

The measured amplitude of respiratory-induced diaphragm motion on 4DCT was on average larger than the 6–17 mm range reported in literature [15, 16, 32]. However, patients in our cohort had an older age at treatment (mean 14.5 years, range 8.6–17.9 years) than those in other studies (ranges 1–20 years), and we excluded a patient treated under GA. Two studies divided their cohort into 2 groups based on age (> 9 years); when we compared our results to their older age groups (*n* = 9; mean 12.3 years [15] and *n* = 18; mean 15.3 years [32]), we saw a similar range of diaphragm motion. Although different ranges of diaphragm motion have been found for younger versus older children [15, 32], no clinically significant correlation has been found in studies investigating possible relationships between respiratory-induced diaphragm motion and age [14, 15]. The same holds for patients treated under GA; differences in amplitude of respiratory-induced diaphragm motion in patients treated with- or without GA were insignificant [14, 32].

Outcomes reported in adult studies that investigated the predictive value of measurements done in the 4DCT are not consistent; some studies found that measurements in 4DCT did not accurately predict respiratory-induced motion as seen on daily/weekly CBCT images [11–13, 18], while others concluded that respiratory-induced motion as measured in 4DCT was representative for the daily motion during the treatment course [24, 34]. These differences mostly depended on the tumor location, considering that abdominally located tumors could also be affected by abdominal processes, while thoracically located tumors are situated closer to the mediastinum. Interestingly, in those adult studies where respiratory-induced motion measured in the 4DCT was not representative, measurements overestimated daily respiratory-induced motion [11, 13], while in our pediatric cohort, the 4DCT mostly underestimated the daily respiratory-induced diaphragm motion. To account for respiratory-induced motion using such a single measurement could possibly lead to insufficient target coverage. Therefore, our results suggest monitoring of respiratory motion with CBCT on a more regular basis, and adapt treatment plans to the actual breathing amplitude when necessary.

For 7 out of 12 patients, the averaged CC positions of the diaphragm during the treatment course differed significantly from the baseline diaphragm position as measured on the 4DCT, introducing a systematic interfractional position variation. The patient number in the present study is low and some patients only had a few CBCTs. This means that measurements regarding baseline-shifts could have been random. However, these results emphasize the benefit and need for daily imaging and monitoring to enable baseline positioning correction.

Nevertheless, present and previous results also confirm that respiratory motion in children varies from day-to-day and even within consecutive breathing cycles [14, 17]. The measured respiratory-induced diaphragm motion on CBCTs acquired within a 4–10 min interval showed significant differences, meaning that the actual respiratory-induced motion during dose delivery can again be different than measured on the CBCT. However, this analysis was only based on a small number of repetitive CBCTs (*n* = 13) evaluated in six patients. Future studies should involve larger imaging datasets for evaluation of measurements on CBCTs for predicting respiratory-induced motion in children. In addition, as mentioned above, it would be interesting to asses respiratory-induced motion online using CBCTs acquired at, for example, the first three treatment fractions. This would enable to identify which patients deviate from their pre-treatment measurements on 4DCT and might benefit from an adaptive approach in order to maintain appropriate tumor dose coverage.

## Conclusions

In conclusion, respiratory-induced diaphragm motion in children determined on 4DCT does not accurately predict the daily respiratory motion observed on CBCTs, as the amplitude differed statistically significantly in the majority of patients. Our results show the limitations of using a single pre-treatment 4DCT to take the patient-specific respiratory-induced diaphragm motion for treatment planning purposes into account. Regular monitoring of respiratory motion during the treatment course using CBCTs could yield a higher accuracy when a daily adaptation to the actual breathing amplitude takes place.

## Additional file

Additional file 1: **Figure S1.** A_CBCT_ values (black dots) plotted as function of days (day 0 is the day of 4DCT acquisition). Lines are linear fits to the A_CBCT_ data; slopes (mm/day) are indicated in the legends next to the dotted line symbol. A_4DCT_ values (open dots) were not included in the fit. (DOCX 201 kb)

## Abbreviations

3DCT: 3-Dimensional computed tomography
4DCT: 4-Dimensional computed tomography
4DMRI: 4-Dimensional magnetic resonance Imaging
A: Amplitude
ALARA: As low as reasonably achievable
AS: Amsterdam Shroud
CBCT: Cone beam computed tomography
CC: Cranial-caudal
CT: Computed tomography
CTV: Clinical target volume
eNAL: extended no-action level
GA: General anaesthesia
IM: Internal margin
ITV: Internal target volume
MR: Magnetic resonance
OAR: Organ at risk
PTV: Planning target volume
SD: Standard deviation
