# Supplementary material for: Predictive value of pediatric respiratory-induced diaphragm motion quantified using pre-treatment 4DCT and CBCTs
Source: Radiat Oncol. 2018 Oct 11;13:198. doi: 10.1186/s13014-018-1143-6 (PMC6180457; doi:10.1186/s13014-018-1143-6)
Supplement: Supplementary file 1 — Figure S1. ACBCT values (black dots) plotted as function of days (day 0 is the day of 4DCT acquisition). Lines are linear fits to the ACBCT data; slopes (mm/day) are indicated in the legends next to the dotted line symbol. A4DCT values (open dots) were not included in the fit. (DOCX 201 kb) [file 13014_2018_1143_MOESM1_ESM.docx]

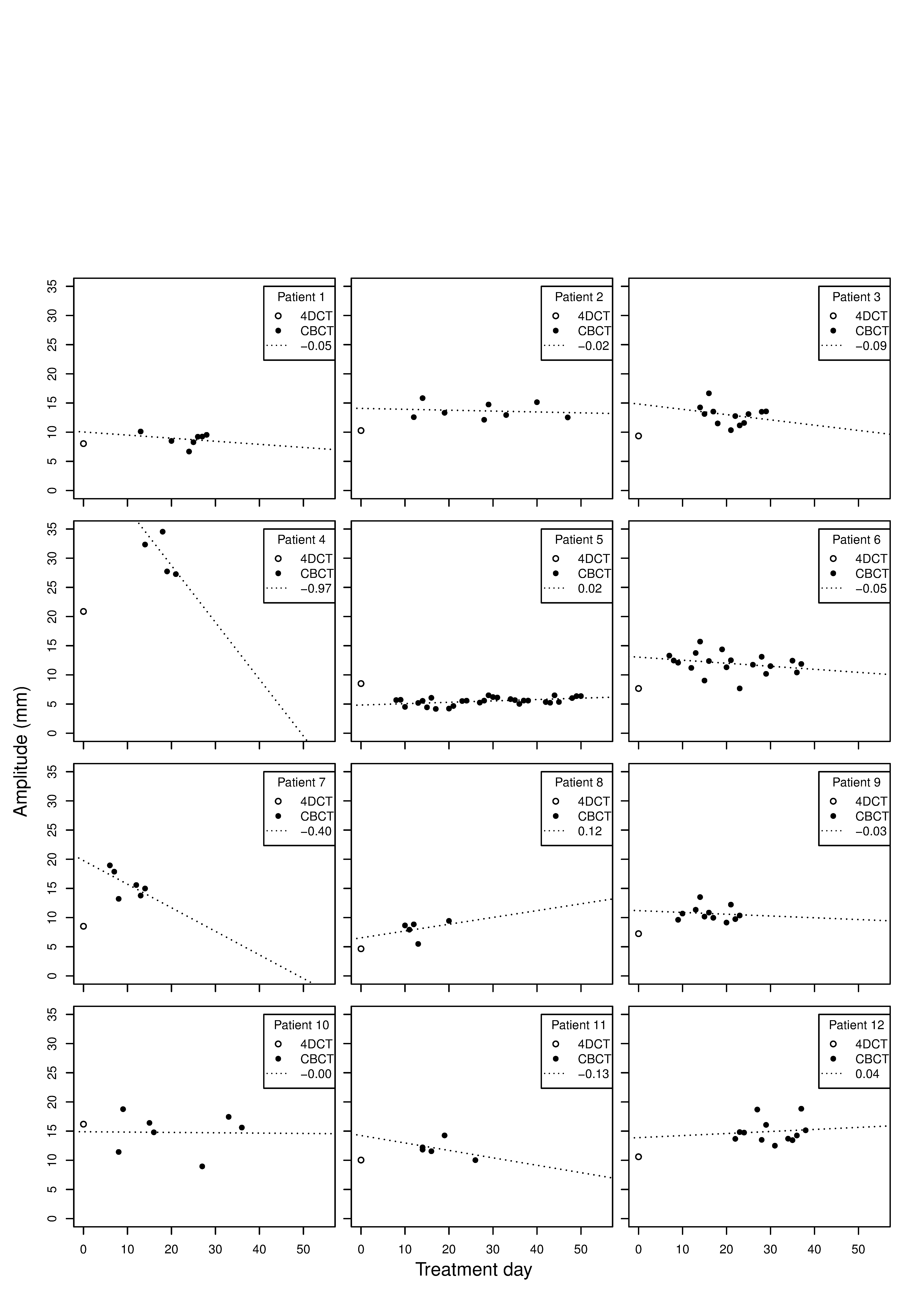


Additional file 1: **Figure S1**. A_CBCT_ values (black dots) plotted as function of days (day 0 is the day of 4DCT acquisition). Lines are linear fits to the A_CBCT_ data; slopes (mm/day) are indicated in the legends next to the dotted line symbol. A_4DCT_ values (open dots) were not included in the fit.
